# The Oxytocinergic System as a Mediator of Anti-stress and Instorative Effects Induced by Nature: The Calm and Connection Theory

**DOI:** 10.3389/fpsyg.2021.617814

**Published:** 2021-07-05

**Authors:** Patrik Grahn, Johan Ottosson, Kerstin Uvnäs-Moberg

**Affiliations:** ^1^Department of People and Society, Swedish University of Agricultural Sciences, Alnarp, Sweden; ^2^Department of Animal Environment and Health, Section of Anthrozoology and Applied Ethology, Swedish University of Agricultural Sciences, Skara, Sweden

**Keywords:** stress reduction, oxytocin, nature archetypes, nature-based rehabilitation, health promotion, vitality, restorative, biophilia

## Abstract

Ever more research results demonstrate that human health and wellbeing are positively affected by stays in and/or exposure to natural areas, which leads, among other things, to a reduction in high stress levels. However, according to the studies, these natural areas must meet certain qualities. The qualities that are considered to be most health promoting are those that humans perceive in a positive way. Theories about how natural areas can reduce people’s stress levels and improve their coping skills have mainly focused on how certain natural areas that are perceived as safe reduce the activity of the hypothalamic-pituitary-adrenal axis and consequent reduction of cortisol levels. This article discusses studies containing descriptions of how participants in rehabilitation perceive and react to natural phenomena. The common core variable in the analyzed studies was the experience of calm and connection, and this experience was associated with a reduction in stress levels and with being able to develop health and coping skills. We suggest that this experience provides a possible role for the oxytocinergic system to act as a physiological mediator for the positive and health-promoting effects in humans caused by nature. The theory is mainly based on analogies framed by theories and data from the fields of environmental psychology, horticulture, landscape architecture, medicine, and neuroscience. Oxytocin promotes different kinds of social interaction and bonding and exerts stress-reducing and healing effects. We propose that oxytocin is released by certain natural phenomena experienced as positive to decrease the levels of fear and stress, increase levels of trust and wellbeing, and possibly develop attachment or bonding to nature. By these effects, oxytocin will induce health-promoting effects. In situations characterized by low levels of fear and stress in response to release of oxytocin, the capacity for “growth” or psychological development might also be promoted. Such an instorative effect of nature, i.e., the capacity of nature to promote reorientation and the creation of new coping strategies, might hence represent an additional aspect of the oxytocin-linked effect profile, triggered in connection with certain nature phenomena. We conclude by proposing that the stress-relieving, health-promoting, restorative, and instorative effects of nature may involve activation of the oxytocinergic system.

## Introduction

All over the world, cities are growing and taking up more and more land. The increasingly large-scale urbanized areas thus need to function for many purposes, not least recreational. Research shows that activities in and/or exposure to certain types of nature and garden areas do affect human health positively, especially when it comes to reducing stress levels and rehabilitating people affected by stress-related mental illness (Egorov et al., 2016; Grahn et al., 2017; Soga et al., 2017; Twohig-Bennett and Jones, 2018; Nilsson et al., 2019). As regards planning, design, and management of urban green areas, the need to be able to classify the areas in an adequate manner thus increases, so that the classification contains both a description of the area and its significance for human health and wellbeing. The designers of urban green areas and therapeutic gardens have adopted and used information from research for evidence-based design (Bengtsson and Grahn, 2014; Stoltz and Grahn, 2021; Vujcic Trkulja et al., 2021). For many years, the research and development work focused on descriptions of content in parks and nature areas that attract visitors. This usually resulted in fairly detailed and long descriptions of, for example, paths, park benches, flower plantations, and play equipment (e.g., Mertes and Hall, 1996). Later, attempts were made to find a link between the content of green areas and health outcomes (e.g., Cooper Marcus and Barnes, 1999), but the descriptions of the content of green areas were still very long and detailed. Later, methods have been developed in order to generally describe properties in green areas that affect human health, such as the perceived sensory dimensions (PSDs; Grahn and Stigsdotter, 2010; van den Bosch et al., 2018; Stigsdotter et al., 2020). The PSDs describe green areas based on whether people perceive them to be more or less open, sheltered, cohesive, diverse, tranquil, etc. These descriptions work well in many contexts around the world and can also be linked to human health and wellbeing (e.g., Lindholst et al., 2015; Mansor et al., 2017; Memari et al., 2017; Vujcic and Tomicevic-Dubljevic, 2018; Chen et al., 2019). However, these PSDs are not related to the specific conditions in a place regarding, e.g., certain animal and plant species. In addition, and more importantly, PSDs cannot be linked to the more worrying and frightening parts of nature. Thus, the contrasts of nature cannot be understood in all respects how they can support a person’s recovery or rehabilitation. They would therefore need to be supplemented with a different type of description.

Research studies assert that people are born with innate preferences (Voland and Grammer, 2003; Galetta, 2014), and this applies not least to natural areas (Falk and Balling, 2010; Ross and Mason, 2017). In addition, research suggests that humans have innate abilities regarding how to select information in order to behave appropriately in different natural environments (Yorzinski, 2014; Coss and Penkunas, 2016). These facts and hypotheses are close to C.G. Jung’s concept of archetypes. These are defined as universal, archaic attributes of an object, a situation, or a context: characteristics that are assumed to be linked to human innate knowledge or readiness, which suggests and/or governs behavior (Sharp, 1991). Archetypes-as-such are diffuse, deep-seated hidden forms in the collective unconscious. From these hidden forms emerge images and motifs, such as the flood, the great mother, the shadow, and others. These images are affected by the context in which they are created (country, culture, etc.), and can thus appear different (Stevens, 2006). Jung’s theory was that some strong archetypes have been found in all cultures since time immemorial. Archetypes can symbolize aspects of the ego (such as persona, anima/animus, the shadow, and the self); monumental legends (such as the creation, the flood, and the apocalypse); important events in human life (such as birth, death, and marriage); or archetypal characters (such as the great mother, the great father, the child, the devil, the wise old man, the wise old woman, the trickster, the hero, the maiden, and the animal; Sharp, 1991). Natural environments have been a part of human beings throughout evolution, so in that sense, there should be strong archetypes related to natural environments. Certain archetypes, such as creation, the flood, the apocalypse, and the animal, of course include nature and natural environments, and appear in many religious legends and myths. Jung suggested no other specific nature archetype, although numerous myths and legends are linked to certain types of natural areas (Sharp, 1991). Sabini (2008) argues that C.G. Jung was close to suggest nature archetypes that were associated with mythological figures, archaic religions, and gods that are closely associated with natural environments and phenomena, such as the sun and thunderstorms.

Bourassa (1988) suggested that Jung’s concept of archetypes could become a basis for a theory in landscape architecture and landscape planning. Today, several researchers claim that in landscape planning and landscape architecture, it is necessary to capture and describe a holistic meaning and content in the landscape, and suggest that archetypes are the solution (e.g., Wilkinson, 2015; Olszewska et al., 2018; Xing and Chen, 2018; Eisenack et al., 2019; Evers et al., 2019; Sietz et al., 2019). Moreover, many theories that attempt to explain the health effects of staying in and/or engaging with natural environments are based on evolutionary hypotheses: They assume that human roots or ancient “homes” exist in certain natural landscapes (Adevi, 2012; van den Bosch and Bird, 2018). Health-promoting properties in natural areas are, for example, suggested to be linked to a possible human primeval home on the savannah (Orians, 1986; Summit and Sommer, 1999).

Recently, Ottosson and Grahn (2021) reported the results of a questionnaire study in Sweden of different types of plant and animal species, lakes, mountains, weather phenomena, etc. This resulted in ten clusters, which were interpreted to be ten nature archetypes (Ottosson and Grahn, 2021). They suggested that these nature archetypes may affect people’s preferences, behaviors, and health. The clusters were named the Death, the Storm, the Adventurer, the Sun, the Moon, the Path, the Eternity, the Fertility, the Guardian, and the Garden of Eden. These nature archetypes can, e.g., be related to the great father, the great mother, and the animal (the Sun, the Eternity, and the Guardian), to archaic deities (the Storm and the Adventurer) and not least to important events in human life, such as death and birth (the Death and the Fertility). Some of these nature archetypes convey security and calmness, while other archetypes are more demanding and insecure. The explanation given today about how nature affects human health and wellbeing in a positive direction is about how certain natural areas that are perceived as safe reduce the onset of cortisol, and reduce neurophysiologic activation, including hypothalamic-pituitary-adrenal (HPA) axis activity (van den Bosch and Bird, 2018). We want to introduce an alternative hypothesis, based on understandings from the physiology of oxytocin, suggesting that natural areas that are perceived as safe, calm, familiar, and attractive stimulate oxytocin release, which in turn exerts powerful anti-stress effects (Uvnäs-Moberg et al., 2005). Enhanced activity in the oxytocin system is linked to acute and long-term health-promoting and restorative effects. An overview of the oxytocinergic system is presented below.

Oxytocin is an archaic polypeptide which exists with some structural changes in mammals, birds, fish, and reptiles (Acher et al., 1995). Oxytocin-like peptides have also been demonstrated in plants (Koehbach et al., 2013). In mammals, oxytocin is produced in the supraoptic (SON) and the paraventricular nuclei (PVN) in the hypothalamus and is released into the circulation *via* the posterior pituitary and into the brain from oxytocinergic nerves, extending from the PVN and from axon collaterals from nerves projecting from the SON to important regulatory areas in the brain. Oxytocin of peripheral and central origin may be released in parallel, and together the peripheral and central effects of oxytocin constitute the oxytocinergic system (Uvnäs Moberg et al., 2019).

Oxytocin was originally described as the hormone that stimulates uterine contractions during birth and milk ejection during breastfeeding but has later on been shown to have a multitude of psychological and physiological effects of more general character: It stimulates social interaction, facilitates bonding between individuals, exerts powerful anti-stress effects, and also exerts restorative/healing functions, including stimulation of growth (Uvnäs-Moberg et al., 2015). The entire psychophysiological effect pattern induced by oxytocin has been labeled “the calm and connection system” (Uvnäs-Moberg et al., 2005).

Regarding humans, the ability of oxytocin to stimulate social interaction of various kinds has received the most attention. When endogenous oxytocin is released, various aspects of social interaction between individuals are enhanced. Individuals with high levels of plasma oxytocin have been demonstrated to be more socially interactive when compared to those with low levels, and the sensitivity and reciprocity during interaction are more pronounced. Further, in support of an active role of oxytocin in stimulating social interaction in humans, administration of exogenous oxytocin as a nasal spray has been shown to exert positive effects on social interactive skills in autistic individuals and to decrease anxiety in individuals with anxiety disorders and post-traumatic stress disorder (PTSD; Tops et al., 2014; Auyeung et al., 2015; Andari et al., 2016; Feldman et al., 2016; Shamay-Tsoory and Abu-Akel, 2016; Frijling, 2017).

In addition, oxytocin decreases stress levels, by decreasing the activity in the HPA axis as well as by decreasing the function in the sympathetic nervous system, thereby lowering cortisol levels, blood pressure, and heart rate. In contrast, oxytocin increases the activity in the parasympathetic nervous system, which, e.g., leads to increased heart rate variability (HRV) and stimulation of digestive, anabolic, and restorative functions (Uvnäs-Moberg et al., 2015). Moreover, oxytocin exerts anti-inflammatory effects by a multitude of actions. It, e.g., decreases the levels of proinflammatory cytokines and the levels of oxidative stress. Oxytocin also stimulates the activity of the immune system, e.g., T cells (Buemann and Uvnäs-Moberg, 2020). Oxytocin release is stimulated by hormonal (e.g., estrogen) and neurogenic effects. The most well-known examples of neurogenic pathways, which are linked to oxytocin release, are the Ferguson reflex and the suckling reflex induced during birth and breastfeeding, respectively. However, pleasant or non-noxious stimulation of sensory nerves in the skin is also linked to oxytocin release (Stock and Uvnäs-Moberg, 1988). Furthermore, physical stimuli, such as warm temperature applied to the skin, may trigger oxytocin release (Uvnäs-Moberg et al., 1993, 2020b). Additionally, visual and auditory stimuli, such as a friendly facial expression or tone of voice, can trigger oxytocin release and effects as a part of prelinguistic communication (Domes et al., 2014).

Evaluation of the surrounding is performed in the prefrontal cortex and may in case of perceived danger lead to an activation of the amygdala-hippocampal system. In this way, cues regarding stressful situations may induce the feeling of anxiety. They may also increase stress levels as the noradrenergic system in the brain stem will be activated and consequently the activity of the HPA axis and the sympathetic nervous system.

When oxytocin levels are high, the activity of the stress system is inhibited or attenuated, both by decreasing the activity in the amygdala and thereby the sensitivity to stressful stimuli, and by directly decreasing the activity of the HPA axis and of the sympathetic nervous system. The activity of the oxytocinergic system is increased in response to subtle sensory stimulation, e.g., by touch, warmth, and light pressure of the skin, by visual and auditory cues, as well as by mental images depicting peaceful and pleasant situations (Uvnäs-Moberg et al., 2005, 2015). Thus, all these situations should be linked to activation of the oxytocinergic system, including the powerful anti-stress effects.

Oxytocin release does not have to be linked to interaction between humans. Research has shown that interaction between humans and animals is also linked to oxytocin release. Furthermore, the acute and long-term anti-stress and health-promoting effects of human animal interaction have been suggested to be due to a sustained activation of the oxytocinergic system (Beetz et al., 2012).

Given the information above, it is not farfetched to assume that exposure to certain types of nature, or nature archetypes, is likely to be associated with oxytocin release and an oxytocin-linked effect spectrum. It is possible that certain primitive or archaic sensory cues induced while staying in nature activates the oxytocinergic system. Oxytocin could thus be released in response to nature archetypes signaling beauty, pleasure, and safety and consequently give rise to a lowering of the levels of fear and stress. Such an effect of nature is supported by data showing that staying in or being exposed to certain types of nature is linked to decreased levels of cortisol and of inflammation (Payne and Delphinus, 2018; Twohig-Bennett and Jones, 2018; van den Bosch and Bird, 2018; Wen et al., 2019), and effects that may be secondary to activation of oxytocin release.

## Aims and Objectives

Research has suggested that humans interpret phenomena in nature based on deep-seated archetypes-as-such in our collective unconscious. Our hypothesis is that this simultaneously activates psychophysiological systems, including basic emotional experiences and corresponding psychophysiological reactions. These types of psychophysiological reaction patterns are to a great extent controlled from limbic parts of the brain, e.g., the hypothalamus. Two important examples of such systems are the stress and anti-stress systems. The stress system includes the HPA axis which regulates cortisol levels and the sympathetic nervous system. The anti-stress system or the oxytocinergic system involves not only the oxytocin system but also the parasympathetic nervous system and is linked to stimulation of social interaction, decreased stress levels, and stimulation of restorative processes and growth.

Emotions of fear and stress and of wellbeing and relaxation, respectively, occur together with their respective physiological reactions, and in this way, two opposite psychophysiological patterns are created, the fight and flight system and the calm and connection system (Uvnäs-Moberg et al., 2005). In this article, we will focus on the possible role of the oxytocinergic system as a possible mediator of positive and pleasant effects induced by nature, which give rise to wellbeing, relaxation, anti-stress, and restorative/healing effects, i.e., activation of the calm and connection system. We further theorize that activation of the oxytocinergic system in the context of nature may in the long term lead to improved wellbeing and health and that it will also facilitate the development of restorative and instorative effects.

## Materials and Methods

### Methodological Considerations

For several decades, our research group has investigated how humans are affected by being exposed to different types of natural environments. The research group consists of expertise from several different subject areas, such as landscape architecture, horticulture, environmental psychology, physiology, and medicine. The impact arises in different ways, both immediate and in a more protracted way, e.g., as health-promoting or instorative effects, where the latter effects are poorly explained theoretically. Recently, studies have suggested that the natural phenomena that affect humans can be explained as archetypes. Therefore, we wanted to create a hypothesis by which nature-linked archetypes might be connected to activation of archaic psychophysiological systems. In this theory development, we selected studies that might contribute to the knowledge regarding the connection between these systems.

Theory development is a central part of research. Torraco (2005) defines theory development as a research process that is intended to create theory. According to Torraco (2005), researchers in this discipline usually approach their creative work from different paradigmatic perspectives and use their preferred strategies and methods to develop new theoretical knowledge. Their work reflects different philosophical values and assumptions about what constitutes knowledge (epistemology), the essence of being or existence (ontology), and what constitutes value (axiology). Torraco (2005) describes a number of methods that have been shown to give good results. There are methods based on quantitative research (such as Dubin’s Theory Development Methodology), qualitative research (such as Grounded Theory Research), and mixed methods (Theory Development from Case-Study Research and Theory Development through Metatriangulation). Our focus was mixed methods, where we would partly start from a comprehensive quantitative study and partly from case studies. Dooley (2002) regards case studies to provide particularly great opportunities for researchers to develop theories. The unique potential of case study research lies in the opportunity it gives the researcher to observe phenomena from several perspectives. Research *via* case studies has the ability to embrace several cases, quantitative as well as qualitative data, and to embrace several research paradigms.

Westhues et al. (2008) describe the strategies they used for theory development in an interdisciplinary team when working in a mixed methods research program. The goal was to synthesize the results of four subprojects. These included a literature review, qualitative interviews, a quantitative questionnaire survey, and case studies. Initially, they worked mainly through induction, to increasingly turn into abduction which eventually led to a synthesis. In the inductive phase, the researchers needed to gather information and then additional information against saturation, to establish the conditions under which a theory is sustainable or unsustainable. This strategy is iterative between theory and data. The iterative approaches in the method are increasingly approaching a theory, leading to abduction, to design a hypothetical pattern that can explain the case, a proposal for a theoretical structure (Kennedy and Thornberg, 2018). In the final synthesis, the proposal for theory will be created.

Several researchers emphasize that although systematic quantitative data create the basis for a theory building, it is anecdotal case study data that make it possible to finally create the theory (Jik, 1979; Mintzberg, 1979; Dooley, 2002; Torraco, 2005; Westhues et al., 2008). Theory building requires extensive description. Hard quantitative data can reveal different types of associations, but soft data can explain them. This in no way diminishes the importance of using quantitative data. In metatriangulation, quantitative and qualitative data function as validation and control systems. Research using metatriangulation and multiple-paradigm research generally requires a research team. Eisenhardt (1989) pointed out that the use of several researchers offers two advantages. First, several researchers, especially if they have different backgrounds, often have complementary insights that increase the richness of the data, and their different perspectives increase the likelihood of benefiting from any new insights that may be hidden in the data. Experienced researchers can also argue based on their knowledge of the field by referring to their own studies and extensive literature. Second, the convergence of conclusions from several researchers increases confidence, while conflicting views prevent the group from reaching a consensus on the conclusion prematurely.

We also find it of importance to state that the hypothesis presented in this paper, i.e., that oxytocin is involved in the positive effects of nature, be it nature archetypes or other sensory cues, is formulated by three senior researchers who have collaborated for more than 20 years and thus have had time to discuss, understand, and integrate each other’s expertise knowledge. The first author is a professor and expert in landscape architecture, biology, and environmental psychology, and the second author is an expert in horticulture and environmental psychology, whereas the last author is a medical doctor and professor of physiology, who has spent almost 40 years studying oxytocin. During the many years of fruitful discussions, we have experienced the possibility of describing a bridge between the effects by nature on human emotions and physiology and those induced by oxytocin.

It is of importance to state that there are many recent studies describing the effects of administration of oxytocin as a spray on human behavior and emotions and that the results of these studies are not fully comparable with the effects induced by endogenous oxytocin released in connection with physiological stimuli. Oxytocin administered intranasally does not always reach all specific receptor sites and in the correct concentration as does oxytocin released from nerves within the brain. Furthermore, data presented in the literature regarding plasma levels of oxytocin must be interpreted with great caution as the methods used differ as to sensitivity and specificity. Data obtained with RIA generally give lower values than those obtained with ELISA. Furthermore, the pattern of effects sometimes differs substantially indicating that the two techniques may detect different variants of oxytocin (Uvnäs Moberg et al., 2019, Uvnäs-Moberg et al., 2020a).

The paper is primarily written from the perspective of landscape architecture, horticulture, and environmental psychology, and not primarily from the perspective of physiology. For this reason, the terminology may sometimes be confusing to representatives from different specialties. For example, the term restorative has different connotations within the field of environmental psychology where it denotes recovery of diminished daily functions and capabilities (Han, 2018), and within the field of physiology, where restoration has a more basic function meaning healing, e.g., of tissues.

### Included Studies

We chose to include three studies/groups of studies in this specific review for theory development. The first study to be reported in this article is a quantitative study which has resulted in a cluster analysis. The cluster tree, the dendrogram, has thereafter been interpreted qualitatively (Ottosson and Grahn, 2021). The second study to be reported is an introspective study of rehabilitation after a serious traffic accident (Ottosson, 1997, 2001, 2007). The third study presented is a summary analysis of the results of several case studies in Alnarp Rehabilitation Garden. The research group has been active in this laboratory since it was established during the years 2000–2002.

#### Quantitative Questionnaire Study Regarding Experiences of Natural Environments: The Ten Nature Archetypes

This study aimed to investigate whether people group objects and phenomena in nature into special categories that can be identified as archetypes. It is a quantitative interview study in southern Sweden. A total of 547 respondents were included, of which 454 women and 92 men (one missing data). Sixty of these were born abroad, but most of them had grown up in or lived in Sweden for a long time. Thirty respondents were born in one of the Nordic countries, twenty-two in the rest of Europe, and seven in countries outside Europe. Of those born in the Nordic countries, most came from Finland (17), followed by Denmark (9), and Norway (5). Of the respondents who came from the rest of Europe, most came from Poland (9) and the former Yugoslavia (7). The respondents filled in a comprehensive questionnaire, which contained 257 objects and phenomena in natural areas, and the results were processed through Ward’s hierarchical cluster analysis. The results showed that the respondents grouped objects and phenomena in nature into ten distinct clusters, which could be interpreted as ten archetypes (Ottosson and Grahn, 2021).

#### Rehabilitation After a Traffic Accident: Facing Nature During Rehabilitation

A case study describing a difficult rehabilitation after a traffic accident. An important part of the rehabilitation took place in the meeting with various natural areas outside the rehabilitation hospital Orup in Skåne, southern Sweden. The method was introspection, self-observation and self-report (Radford, 1974). In this article, a selection from this introspective study will be recounted (Ottosson, 1997, 2001, 2007).

#### Rehabilitation in a Healing Garden: Experiences From Alnarp Rehabilitation Garden

The data consist of a number of narratives and experiences from patients who have been rehabilitated in Alnarp Rehabilitation Garden, which is a laboratory and a research infrastructure at the Swedish University of Agricultural Sciences in Alnarp, southern Sweden (Stigsdotter and Grahn, 2003). Several hundred people who have suffered from severe life crises, depression, and exhaustion have been rehabilitated at this research infrastructure from 2002 to 2012. The rehabilitation has been followed by researchers, who have used both qualitative and quantitative methods (Grahn et al., 2010, 2017).

#### Synthesis of Data and Development of Theory

Researchers from several disciplines participate in the study: horticulture, landscape architecture, environmental psychology, and medicine with an endocrinological focus. The researchers have extensive experience through several decades of research work in their respective subjects. The above studies are included as data, as well as own background knowledge in the field, including own studies and knowledge of relevant literature, including case studies, case-control studies, and RCT studies including results from measured outcomes regarding blood pressure, heart rate, saliva cortisol, and oxytocin, as well as qualitative interviews, observations, and diaries. The interpretation takes place through induction and abduction in an iterative process toward a final synthesis by consensus. The study triangulates the above data. It is a holistic, reflective research process to bridge all theories, paradigms, data, and results and create a theory development (Dooley, 2002; Torraco, 2005; Westhues et al., 2008).

## Results

### Study One: The Ten Nature Archetypes

**Figure 1:**
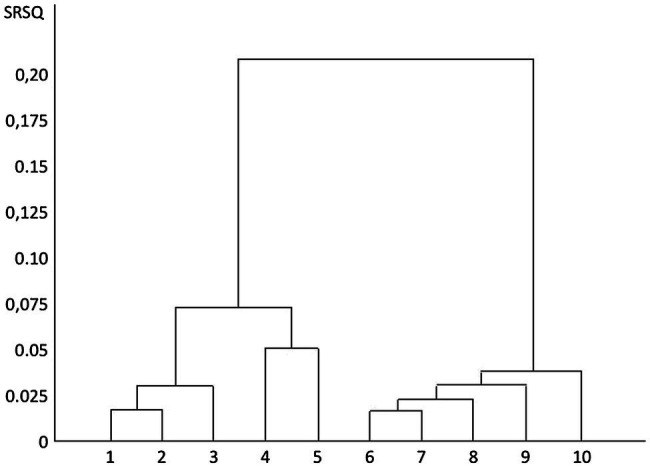
Cluster dendrogram. The dendrogram is based on the participants’ preferences for different qualities in nature. Y-axis semipartial R-square; X-axis clustered preferences. The higher up in the dendrogram: a cluster, or a couple of clusters, has separated from the others, the more they differ from the others. (1) The Death, (2) The Storm, (3) The Adventurer, (4) The Sun, (5) The Moon, (6) The Path, (7) The Eternity, (8) The Fertility, (9) The Guardian, and (10) The Garden of Eden.

The results from the survey with questionnaires are presented briefly below. For a fuller description, see Ottosson and Grahn (2021). Figure 1 shows the dendrogram over the cluster analysis. The main result of the cluster analysis is the cluster tree, the dendrogram, which can be compared to a tree that is upside down; i.e., it has the branches down. At the tip of each of the ten branches is a distinct cluster: a nature archetype. The two large branches divide high up in the dendrogram, and the nature archetypes on these thus differ greatly from each other. The closer the smaller branches in the dendrogram are to each other, the greater the relationship between the nature archetypes.

To start with, the figure shows two distinctly spaced main branches, with five clusters in each. The left main branch is further down divided into two distinct parts, with three nature archetypes to the left and two to the right. The right main branch divides further down, suggesting that the nature archetypes contained here are not as clearly separated. The right main branch divides with one nature archetype to the right and four to the left.

The left main branch was interpreted to contain nature archetypes with demanding natural environments, while the right main branch was interpreted to contain nature archetypes with peaceful, restorative, and recreational natural environments.

The cluster on the far left (No. 1) is interpreted as the nature archetype the Death. It contains animals, such as wolves, ravens, bats, frogs, and spiders. The landscape consists of desolate swamps and dead trees in veils of mist. There are no living plants in the cluster apart from death caps. The second cluster (No. 2) is interpreted as the nature archetype the Storm. It contains a variety of dangerous types of storms, thunder, hail, and heavy rain. The third cluster (No. 3) is interpreted as the nature archetype the Adventurer. It contains, e.g., mountains, rivers, rapids, rafts, and campfires. The fourth cluster (No. 4) is interpreted as the nature archetype the Sun. It contains for instance sunshine, blue sky, and sandy beaches as well as castles, barbed wire fencing, crowds of people, new roads, and motorboats. The fifth cluster (No. 5) is interpreted as the nature archetype the Moon. It contains variables, such as moonlight, night sky, snow, frost, cemeteries, silence, coniferous forests, and older farms with animals.

The sixth cluster (No. 6) is interpreted as the nature archetype the Path. It contains, e.g., hiking trails, cairns, rolling landscapes, brooks, and open landscapes mixed with forests with deciduous trees. The seventh cluster (No. 7) is interpreted as the nature archetype Eternity. It contains many signs of seasonal changes and water, such as melting ice in spring, cracking leaves, and autumn leaves, but also waves rolling toward the beach and big stones. The eighth cluster (No. 8) is interpreted as the nature archetype Fertility. It contains many types of cultivated fields, as well as the orchard and kitchen garden. The ninth cluster (No. 9) is interpreted as the nature archetype the Guardian. It contains likable wild animals, such as hedgehogs, squirrels, hares, deer, and small birds. The tenth cluster (No. 10) is interpreted as the nature archetype the Garden of Eden. It contains, e.g., fruit trees, flowers, hammock, arbor, a pond, water lilies, and butterflies.

The five nature archetypes the Death, the Storm, the Adventurer, the Sun, and the Moon differ substantially from the Path, the Eternity, the Fertility, the Guardian, and the Garden of Eden.

#### Our Interpretation and Conclusion

Through the cluster analysis, 257 objects and phenomena in natural areas have been placed in very clear groups. The nature archetype gives sharp messages about danger or safety, but also about more subtle archetypal messages concerning the passage of time; hope or consolation; adventure and freedom; work and struggle; or rest and reward.

### Study Two: Facing Nature During Rehabilitation

In a number of articles, Ottosson (1997, 2001, 2007) described how nature helped him in a rehabilitation process. Below are some central sections from these articles. The quotes are selected in order to illustrate how his introspective method allows for the exploration and understanding of nature archetypes. They are about Johan Ottosson’s experiences of rehabilitation after a traffic accident that caused a serious head injury and life crisis, and how natural phenomena in southern Sweden provided necessary help and support in his rehabilitation process. In these introspective studies (Ottosson, 1997, 2001, 2007), some of the clusters/nature archetypes described above can be found.

In January 1991, when Johan was 39 years old, he cycled to work and was hit by a car. He himself has no memory of the accident. Sometime after the accident, he ended up in ward 108 at Orup Hospital, a clinic that specializes in the rehabilitation of people who have suffered brain damage. The following quotes describe Johan Ottosson’s meetings with various natural objects, environments, or phenomena in nature, such as seasons or weather, during the rehabilitation period. The method is based on introspection, in which the researcher himself is the subject of the study and in which attempts are made to present the person’s behavior and reactions as objectively as possible. Johan Ottosson claims that he only for a short time could reflect on how nature could help him during rehabilitation. It was as if a window was open to this interpretation. Today, it had not been possible to do so, and when he was in the middle of the rehabilitation, it was too close to be able to have a perspective on what was happening (Radford, 1974; Ottosson, 2007; Weger, 2018).

#### First Quote

Johan Ottosson remembers that he first walked around the hospital area – short walks near the hospital – but over time it became longer and longer walks out into the landscape (Ottosson, 2007).

The nature around Orup (a hospital in Southern Sweden) pleased him. It gave a wild impression while the traces of man were present. Both the tracks from ancient times and the tracks from new times were there. The traces of old times with the moss-overgrown dry-stone fences, almost completely overgrown house grounds or lichen-covered, feral apple trees - where you could only imagine leftovers of an old garden - gave him a special sense of security and recognition. Inwardly, he saw images from old times with hard-working small-scale farmers and crofters. The human struggle for survival caused feelings that he recognized (Ottosson, 1997).

Referring to Cluster 8: “Fertility.”

#### Second Quote

One of his problems after the accident was the ability of wayfinding. There was always a risk of not being able to find his way back again. The need to be in nature was countered by a fear of getting lost. But this fear did not hinder him, and although he could not explain why, the daily encounters with nature were indispensable (Ottosson, 2007).

The path is created through an interplay between animals/Man and Nature. The path respects the demands Nature makes – it leaves no scars but runs like a natural nerve through the whole. Why the path inspired such feelings he did not know. Perhaps it was some innate feeling of belonging that goes back to our beginnings. Paths have always been our friends – strands leading from one secure point to another. A gift passed from one generation to the next. When he followed paths that he liked especially much, it made no difference where they led – he wished the path would never end. He liked to walk along paths rather than ramble over the terrain, even in familiar territory. Seeing the path before him was a thing of beauty, it touched something deep in his subconscious. This feeling led him to keep to paths, which was perhaps for the best. This predilection has been vital to our survival since time immemorial. What he found beautiful was the way past generations passed the experience to him. This age-old feeling pleased him (Ottosson, 2001).

Referring to Cluster 6: “The Path.”

#### Third Quote

Johan Ottosson preferred to be alone when out in the wild (Ottosson, 2007). The feeling of communion, harmony, and of being able to attune to nature was too subtle, too fragile to compete with the company of other people. And this strong need for solitude in nature was something new for him. Johan Ottosson found it difficult to describe this need and feeling, and could not compare it with anything. When he had company with others, nature assumed a different and more passive role, and the landscape was transformed into a backdrop (Ottosson, 2007).

When he thinks back to the early days, right after the accident, he is surprised by how many of his impressions from the natural surroundings are connected with stones. The untouched stone with its blanket of lichen and moss in various shades of green and gray gave him a sense of security through its timelessness, its calm and harmony. It was as though the stone spoke to him: “I have been here forever and will always be here; my entire value lies in my existence, and whatever you are or do is of no concern to me.” The stones did not speak to him with words, but in feelings, which made the relationship both deep and strong. The feelings calmed him and filled him with harmony. His own situation became less important. The stone had been there long before the first human being had walked past. Countless generations, each with lives and fates of their own, had passed by (…) ideally, he wanted to be alone with the stones and the rock outcrop. The calm atmosphere that an old stone radiates is easily lost. It is as if the stone could absorb the grief. Share it without self being consumed. The tears that end up on a hot rock outcrop evaporate, disappear and part of the grief with it (Ottosson, 1997).

Referring to Cluster 7: “Eternity.”

#### Fourth Quote

Johan Ottosson describes the experience of having suffered brain damage: That it turned his world upside down. Not being able to do things that used to be second nature and which most people take for granted was a scary experience. It was like living in a fantasy world over which he had no control – a nightmare he could not wake up from (Ottosson, 2007). The primordial forces of the wind and the sea attracted him. Maybe it was because the sea gave him an outlet for his rage over his weakened condition. The sea helped him to ban the higher powers for having thrust him into such a situation (Ottosson, 2007).

He liked the sea in all of its moods, but especially in raging storms. He liked walking down to the shore when the wind whipped the sea into a frenzy. The primeval forces of the wind and the sea attracted him. Perhaps it was because the sea gave him a vent for his fury over his weakened condition; with the help of the sea he cursed the gods for having thrust him into such a situation (…) The raw forces of nature appealed to him. In the face of such tremendous power we are all small, helpless creatures. His own situation was not much different from that of others. Face to face with Nature, we are all equal – even the strongest has to give in. While out on his walks he did not feel inferior to anyone (Ottosson, 2001).

Referring to Cluster 2: “The Storm.”

#### Fifth Quote

Coping with a difficult life crisis by getting help to curse the situation is one side of getting consolation and relief from pain; another is to find tranquility and repose (Ottosson, 2007).

The untouched sandy beach, clean and devoid of life, but with the constant motion of the waves, called to him. Once there, he found it hard to leave. The waves that washed over the sand seemed like some eternal pulse, something that had always been there and would always be. The sound and sight of the waves against the sand filled him with calm and a sense of security – like the heartbeat of the mother to an infant child. This feeling was so basic that he could never lose it. That this was so gave him a feeling of security – the element of eternity appealed to him (Ottosson, 2001).

Referring to Cluster 7: “Eternity.”

#### Our Interpretation and Conclusion

Johan Ottosson’s introspective description and narrative provides interesting examples of how natural environments can carry archetypal messages: These contain clear and at the same time quite sensitive and finely tuned conveyed messages about consolation, perspectives in life, paths, and promises. The narrative also shows the importance of nature’s contrasts and dynamics, between the powerful roaring storm and the calm waves on the lake, and that even old house foundations and stones get a different expression when lichen and moss take over. Nature is something that alters, there is a movement that shows life and change. In the midst of all this, there is stability, calmness, and security. The narrative also shows that relations with nature need to take place in solitude and silence, in order for nature’s messages to be clearly conveyed and experienced.

### Experiences From Alnarp Rehabilitation Garden

**Figure 2:**
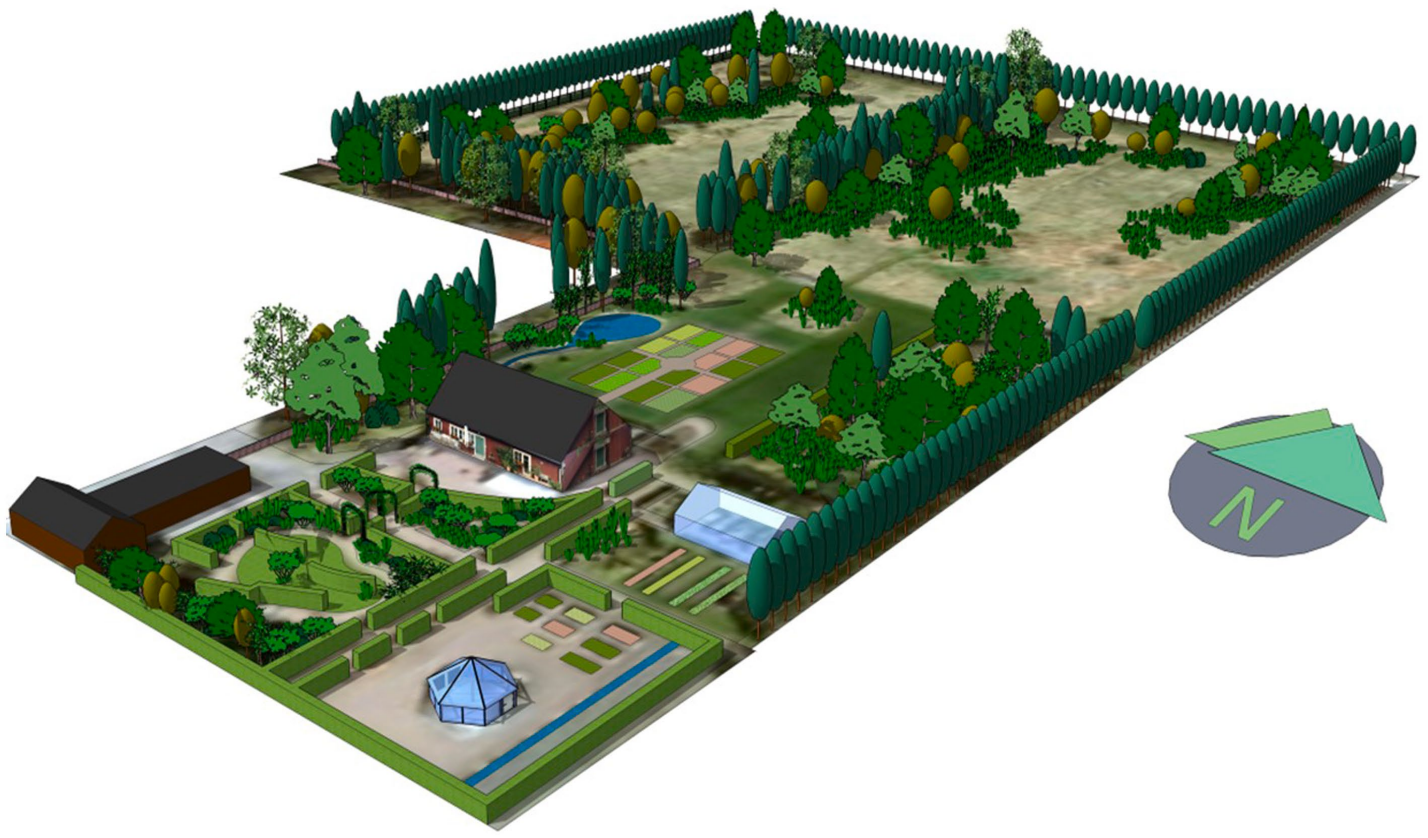
Alnarp Rehabilitation Garden, part of the Swedish University of Agricultural Sciences’ research infrastructure. The garden is approximately two hectares in size and is designed with a number of garden rooms with different designs and contents. Studies of patients in the garden revealed behavioral changes associated with different types of treatments, which may be associated with release or not of oxytocin. The model of the garden is made by Gunnar Cerwén.

Some nature-based therapeutic activities have been thoroughly analyzed over the years, notably at Alnarp Rehabilitation Garden at SLU Alnarp campus area outside Malmö in Sweden (Stigsdotter and Grahn, 2003). These gardens have been designed to contain many qualities (Stigsdotter and Grahn, 2002, 2003; Ivarsson and Grahn, 2010; Sahlin et al., 2012; Pálsdóttir et al., 2018). Researchers who observed the participants have discovered that they move across the garden to look for different places that match and support their emotional state (Ivarsson and Grahn, 2012; Pálsdóttir et al., 2014; Adevi et al., 2018). Some results in Alnarp Rehabilitation Garden (see Figure 2) are interpreted as being characterized by oxytocin-linked effects, which in turn are explained by calming and supportive activities in a safe relaxing garden and natural environment (Adevi et al., 2018).

Researchers found that crisis-stricken participants seemed to be lost at the beginning of rehabilitation. They walked around uncomfortable and uneasy without finding any peace. After a time, however, they felt safe and thus found themselves settled. With that, they withdrew to lush nature areas in the garden, to reflect alone on their situation in peace and quiet (Pálsdóttir et al., 2014). An observational study in Alnarp found that during garden rehabilitation, the participants moved in two specifically different ways. One of these was characterized by the participants walking calmly and introvertedly, as in meditation, straight, and determined. The other was characterized by an unsettled extroverted walk, around the whole garden, picking with plants and trying to talk to other participants (Ivarsson and Grahn, 2012). A later study found that the psychotherapeutic treatment in the garden stirred up feelings that the participants seemed to have difficulty coping with, and they were constantly moving. The physiotherapeutic body treatment, on the other hand, seemed to calm the participants, which led them to find a place in the garden where they could reflect (Adevi et al., 2018). The researchers interpreted the rehabilitation program at Alnarp Rehabilitation Garden with the conclusion that the differently designed garden-rooms and the staff together offered a supportive environment: a safe haven where participants could feel trust and belonging. Hence, it was possible to start a journey where the participants’ coping skills could be strengthened (Grahn et al., 2010; Pálsdóttir et al., 2014), accelerated by an oxytocin release (Adevi et al., 2018). In particular, the physiotherapeutic treatment came to stimulate the release of oxytocin, which was marked by the behavior. Thus, the participants could find a good natural environment where they could reflect on their situation (Adevi et al., 2018).

Often, the participants’ activities are about finding restful, calm places as well as finding places that bring joy, stimulation, and challenges. Such places, usually beautiful, are relatively plentiful in the landscaped healing gardens. However, other places, not as appealing, are also needed. In the northwest corner of Alnarp Rehabilitation Garden, there is a fruit grove, where the trees are affected by fungi and decay. Several trees are dead or dying. At the northernmost edge, dense blackberry thickets grow between the trees and a fence. Several participants specifically visited this place, and one of them states “I swore and cursed and cried and carried on by myself to just be alone and sort of try to let off steam and sort of let out everything” (Pálsdóttir et al., 2014). A few others even made their way through the blackberry thicket, even though the tags tore in clothing, hands, and face. The therapists in the garden said that there were too few of these challenging “malicious” places in the garden (Grahn et al., 2010). Not everyone needs these challenging places, but for some participants, finding them can be crucial to their rehabilitation. Each crisis process has both individual characteristics and generalities.

#### Our Interpretation and Conclusion

The results from this study also show the importance of the participants primarily needing to find places where they can feel peace and tranquility as well as security. They can achieve this if they are allowed to be alone, where they can feel trust and friendship with both themselves and with nature. It is about affinity but does not have to lead to attachment. It is more about to attune and get in harmony, about calm and connection. Staff, here in the form of a physiotherapist, can enhance the calmness, and then, the participants find it easier to find peace in the garden. A psychotherapist who asks provocative but important questions can, on the other hand, lead to participants having to struggle to find their mental balance in the garden. It is not certain that they will then need to look for the most beautiful and calm places. Nature also contains a story of wounds and thorns, death, and transience, which they may have to face and master.

### Synthesis of Studies and Evolution of Key Categories in Theory Development

In the last step, we have combined results and conclusions from the studies, how they converge by means of triangulation and crystallization, and build bridges that can help develop the hypothesis and theoretical model (Dooley, 2002; Torraco, 2005; Westhues et al., 2008).

We found the following four key categories:

#### Key Category 1: Danger or Security

This key category is clearly present in all studies. It emerges particularly clearly as the basic dual archetypal phenomenon and forms an upper hierarchy in the cluster diagram in study one. And it is obvious that the participants in Alnarp Rehabilitation Garden during the first week tried to find places where they felt safer. In study two, Johan Ottosson describes the problem of not being able to feel secure and calm in the hospital – instead unsettled – and that despite problems with being able to find his way back to the hospital, he sought out places in nature where he could find this.

#### Key Category 2: Subtle Archetypal Messages

In addition to the overarching concepts of danger and security, all studies present clear conclusions that nature contains other messages: about perspectives in life, wounds and thorns, death, and transience, hope and comfort, adventure and freedom, work and struggle, or rest and reward.

#### Key Category 3: The Importance of Nature Constantly Changing

All studies report the importance of nature’s inherent power, which involves movements that show life and transformation, contrasts, and dynamics. In the midst of all this, there is stability, peace, and security.

#### Key Category 4: Creating a Sense of Affinity

This key category is very pronounced in the introspective study as well as in the studies in Alnarp Rehabilitation Garden. Basically, it is about being able to build trust and friendship, with both oneself and nature. It is about creating a sense of belonging but does not have to lead to attachment. We call this “affinity and attunement.” This seems to be easier to create if people are alone with nature, in peace and security.

#### Core Variable: Calm and Connection

As the research team continued to compare outcomes with outcomes in the data and built them into categories, a larger category began to emerge. This key category was larger than the others in its abundance of outcomes and associations, and on closer analysis, we found that it had explanatory value for each of the other key categories. In the last step, this fifth key category was transformed into a core variable, as the other key categories came to revolve around it. This core variable seemed to account for most of the variation in what was in focus for the study, and thus became the focus for further discussion (Holton, 2008; Byström et al., 2019). We found that the core variable, which is strongly bound and anchored in all the above key categories, is calm and connection.

## Discussion

The aim was to develop a hypothesis about why and how stays in and/or being exposed to certain natural areas could improve people’s mental wellbeing and health. Moreover, the aim was to develop a hypothesis connecting humans’ interpretation of phenomena in nature based on deep-seated archetypes-as-such in our collective unconscious with archaic psychophysiological systems. In this respect, we have combined results from a quantitative interview study and case studies. In addition, through our background in different research disciplines, we can refer to several studies, our own and others’, as well as literature. We have studied how results and conclusions converge to find bridges that can help develop the theoretical model (Dooley, 2002; Torraco, 2005; Westhues et al., 2008). We found four key categories and a core variable, calm and connection, which is strongly tied and anchored in all key categories. In the end, we make an attempt to link the positive, health-promoting effects caused by stays in nature to activation of and effects of the oxytocinergic system.

Our hypothesis is that experiences of certain nature archetypes simultaneously activate psychophysiological systems, including basic emotional experiences and corresponding psychophysiological reactions. In addition, that the oxytocinergic system is involved in various ways when it comes to people’s relationship with natural areas. On the following pages, we will describe this in more detail. The discussion has three main parts. We start with the key category “danger or security,” which are phenomena that are often highlighted in explanations of how people can recover from stress when exposed to natural areas: Restorative Processes and Environments. We continue with the other three key categories that are more associated with longer periods of processing mental issues: Instorative Processes and Environments. Finally, we deal with the core variable “calm and connection” and describe the theoretical model. We discuss how activation of the oxytocinergic system in response to cues from nature functions as an important physiological mediator of both restorative and instorative processes.

### Restorative Processes and Environments

#### On Innate Preferences Originated Through Evolution

The first key category relates mainly to scientific discussions in environmental psychology belonging to the concept of restoration. Restoration is defined as the recovery of diminished daily functions and capabilities, largely during people’s free time (Han, 2018). Restorative environments are defined, according to von Lindern et al. (2016), as environments that both permit and promote restoration. Several studies have shown that spending time in natural environments is positive for human health (e.g., Egorov et al., 2016; Nilsson et al., 2019). People who suffer from high levels of stress restore their capacities when staying in and/or being exposed to natural environments but not so in built-up environments (Ulrich, 1993; van den Bosch and Bird, 2018). Furthermore, people who suffer from a drained, fatigued directed attention capacity can restore their ability when exposed to natural environments but not so in built-up urban environments (Kaplan, 1995, 2001). However, most of these studies have used extreme types of natural environments and built-up environments: tranquil alluring and bright natural environments against the gray noisy city. Though, it is unlikely that all natural environments will reduce people’s high stress levels (Bixler and Floyd, 1997; Stigsdotter et al., 2017).

Nature archetypes are supposed to be innate and are assumed to guide people regarding typical phenomena and patterns of action that have followed human beings through evolution. The most widely accepted interpretation today of Jung’s archetypes is that they are innate dispositions to detect and react on objects or coherent patterns, the so-called archetypal images or symbols (De Coro, 2018). “The archetype is a tendency to form (…) representations of a motif —representations that can vary a great deal in detail without losing their basic pattern” (Jung, 1964). Since nature has followed humans for millions of years through evolution, patterns of action in relation to typical characteristics and phenomena in nature should have been inherited. We interpret the key category “Danger or security” as basic archetypal phenomena, which can be related to theories and research results regarding biophilia and biophobia.

#### Environments Humans Instinctively Avoid Frightening Environments: Biophobia

The five clusters in the left branch (Figure 1) contain objects and environments that are mostly demanding. In the first two clusters, there are several well-known phobic objects and environments, such as spiders, snakes, wasps, big predators, thick fog, storm, and thunder. In clusters three to five, there are heights, congestion, darkness, and complete silence. These five clusters contain clear examples of the most biophobic environments and objects, used extensively in horror movies and thrillers. It is well known that perceiving certain natural phenomena can quickly and unconsciously trigger a panic reaction in us (Öhman, 2008): the so-called “biophobic” objects, scenery, and events. Our evolutionary history is undoubtedly in the fright and phobias. We are more likely to fear events and situations that were threats to the survival of our ancestors, such as potentially deadly snakes, darkness, and heights, than to fear the most common mortal objects in modern life, such as traffic. From an evolutionary perspective, perceptual systems are biased toward discovering threat (Öhman and Mineka, 2001). This is explained by the fact that, during evolution, humans had to reflexively avoid or flee these phenomena, thus having a greater chance of survival. Öhman (2008) speaks of main categories of phobia: those to do with animals (big predators, snakes, and spiders), social phobias (congestion), and spatial phobias or wayfinding (steep precipices, open spaces, thick fog, and darkness), that is, animals and environments that can be found in the five archetypes in the left part of the cluster. This means that vigilance increases as people approach environments associated with these five archetypes. If danger is signaled, for example, by the presence of a snake, the amygdala is activated and consequently affects linked to fear and the sympathetic nervous system is activated. This trigger, among other things, the activation of the cardiovascular system and the release of a number of hormones, such as cortisol and catecholamines. The entire organism is mobilized for an emergency reaction which has been in place in the brains of mammals for a hundred million years and is of vital importance for survival (Tomkins, 1995; Öhman, 2008).

#### Environments People Actively Seek for Restorative Environments: Biophilia

In the right branch of the cluster tree (Figure 1), there are several environments that can be described as restorative: open landscapes with deciduous forests, hills, and streams in cluster six; appealing and tranquil sounds and scents in cluster seven; groves, meadows, and pastures in cluster eight; friendly animals in cluster nine as well as fruit trees, flowers, and birdsong in cluster ten. It is well known that staying in and/or being exposed to natural environments is linked to restorative stress-reducing effects, such as lowered cortisol levels and blood pressure, and these natural areas are described as having the qualities found in the right part of the cluster tree (Twohig-Bennett and Jones, 2018; van den Bosch and Bird, 2018; Nilsson et al., 2019). Several frequently quoted researchers claim that people through evolution have received higher preferences for particular objects and phenomena in certain natural landscapes. The primeval home of humans has been hypothesized to be located in protective green surroundings: to be restful, without disturbing sounds and smells, and commanding a view of predominantly lightly forested, open fields (Appleton, 1975; Falk and Balling, 2010). We are presumed to prefer these environments because they are associated with a sense of security, and we can therefore recover from high stress levels in these landscapes.

The concept biophilia was first used by the psychoanalyst Fromm (1973), who defined biophilia as “the passionate love of life and of all that is alive.” This term was however later used by Edward O. Wilson in his book Biophilia (Wilson, 1984), where he proposed that the tendency of humans to focus on and to affiliate with nature and other life-forms has, in part, a genetic basis: It is a result of an evolutionary heritage. Later, Roger Ulrich has partially redefined the concept of biophilia through his studies, by claiming that biophilic natural environments are the opposite of biophobic environments. Biophilic natural environments are reflexively interpreted as safe (Ulrich, 1993). People most often prefer open, bright landscapes, which is believed to be due to our development into humans in a savannah-like landscape (Orians, 1980, 1986; Balling and Falk, 1982; Falk and Balling, 2010). Sommer and Summit (1995) argued that we have particularly strong preferences for trees with large canopies, and Coss and colleagues (Coss and Moore, 2002; Coss et al., 2003) that we prefer environments close to water. These environments are expected to be able to quickly reduce levels of stress through emotions/affects, particularly when people experience high stress levels (Ulrich, 1993, 2002). Many studies confirm theories of biophobia and biophilia: Humans have a strong inherent preparedness to respond instantly to concrete threats in natural environments but can also recover quickly if the natural environments are judged to be safe (Ottosson et al., 2015; Braubach et al., 2017; van den Bosch and Ode Sang, 2017; van den Bosch and Bird, 2018).

Some researchers argue, however, that their studies point to results that cannot be unambiguously interpreted based on hypotheses of innate preferences. Jiang et al. (2015) found that the most sparse and flat landscapes were not attractive to respondents. Preferences increased rapidly when the forest became denser, but there was no clear peak at a semi-open savannah landscape. Instead, a wide plateau was achieved, where preferences continued to grow slowly the more the forest densified. Hägerhäll et al. (2018) found no clear universal preference for a savannah landscape: Western respondents seem to prefer the “savannah,” but populations from non-Western, indigenous and primarily rural communities in Colombia, East Timor, Malaysia, and Suriname had higher preferences for landscapes with denser forests, similar to the landscapes in which they lived. Studies by, for example, Home et al. (2010), Adevi and Grahn (2012), and Petrova et al. (2015) found that people’s preferences for natural environments and urban parks could be explained by theories regarding both evolution and experiences acquired since childhood. Balling and Falk (Balling and Falk, 1982; Falk and Balling, 2010) found that younger people had preferences for savannah environments, while the preferences of adults and elderly people more closely resembled their home environments. We suggest that this may be because conceivable innate preferences could be affected by how strongly people become attached to the place where they grow up. This process takes time, which can lead to that environmental preferences becoming more and more similar to the place people are attached to. We return to theories of “place attachment” below.

Nevertheless, people in general prefer open, bright landscapes, which should have a particularly stress-reducing effect. These theories and findings have been supported in several studies (e.g., White et al., 2010; Meireles et al., 2014; Muruka, 2014; Gerstenberg and Hofman, 2016; Hofmann et al., 2017). The Stress Recovery Theory (SRT) seeks to explain why contact with these specific biophilic types of natural environments reduce stress levels in humans (Ulrich, 1993). Ulrich et al. (1991) claim that landscapes with the above characteristics improved the chances of survival in archaic humans, and they reduce stress levels in humans today. More specifically, SRT assumes that positive affect, which occurs during contact with those natural qualities, reduces neurophysiologic activation, including HPA activity to a favorable level (Ulrich, 1993, 2002; van den Bosch and Bird, 2018).

As will be discussed more in detail in a subsequent part of the discussion (see section “Calm and Connection, and Its Relation to the Oxytocinergic System”), we suggest that the anti-stress and restorative effects induced by safe, inviting landscapes discussed by Ulrich (1993) are primarily mediated by an activation of the oxytocinergic system.

### Instorative Processes and Environments

#### A Definition of Instoration and Instorative Environments

We find that the two main branches above (Figure 1) can be related to biophobia and biophilia. We interpret this as being about natural phenomena that trigger rapid reflexive decisions that communicate with basic category affects. These reactions can support behaviors that, for millions of years, helped our species and its predecessors when in danger as well as for quick restoration (Ulrich, 1983, 1993; Tomkins, 1995; Ottosson et al., 2015). We interpret the other three key categories as belonging to the concept of instoration.

In section “Study Two: Facing Nature During Rehabilitation,” Johan Ottosson describes neither the rapid onset of fight-flight response nor any recovery from high stress levels. Instead, he describes how he seeks to find a way to improve his coping strategies. It is about being able to relate to feelings that have to do with security, trust, joy, consolation, sadness, and/or anger in connection with how he can manage his situation. There are many emotions that need to be regulated, and subtle archetypal messages can help, regarding perspectives in life, grief and pain as well as hope and comfort, conveyed by natural phenomena. Johan describes this based on his own situation. The phenomenon is also described in research reports in both wilderness therapy and horticultural therapy (e.g., Pálsdóttir et al., 2014; Conlon et al., 2018). When discussing these kinds of nature-based interventions, researchers claim that certain natural areas contribute to something more than just restoration: They talk about instorative effects and environments (Hartig et al., 1996). The word instorative relates to instauration – meaning an act of instituting or establishing something. Instorative effects are about how nature areas appear to act as catalysts, necessary in accelerating the processing of crises so that reorientation is achieved faster (Stigsdotter and Grahn, 2002, 2003; Grahn et al., 2010). The ten branches (Figure 1) have been interpreted to be ten nature archetypes, and we suggest that they communicate with humans on a more subtle level: That they convey senses that lead to instorative effects.

#### To Be Able to Perceive the Nature Archetype as a Whole That Holds Force and Power

One result of this study is the proposal for a key category that deals with the importance of change in natural landscapes, and nature can largely be described in terms of movement and change. People more easily perceive what is moving: It can be perceived as interesting, carrying information, and even alive. In section “Study One: The Ten Nature Archetypes,” we present ten clusters that were interpreted as ten nature archetypes: The Death, the Storm, the Adventurer, the Sun, the Moon, the Path, the Eternity, the Fertility, the Guardian, and the Garden of Eden. The archetypes we encounter in nature can possibly be perceived as carrying messages, which we record with all senses. There is a difference between how one experiences a landscape in calm weather and when it is storming, when it is bright and sunny or in darkness. Nature is always dynamic and changing. Stern (2010) has developed a theory of vitality, which he defines as “a whole … It is a Gestalt that emerges from the theoretically separate experiences of movement, force, time, space and intention” (Stern, 2010). According to Stern (2010), vitality has its foundation in some kind of movement, and this perception is always accompanied by the four qualities of force, space, time and direction/intention.

We choose to interpret vitality as constituting a constant and basic lived involvement in a person’s lifeworld and in the relationship with the environment: the social as well as the physical (Dahlberg and Drew, 1997; Matthews, 2014; Byström et al., 2019). Stern (1985) claims that the dynamics of our surroundings is of crucial importance in how we experience our environment. It goes without saying that we interpret a person’s intention in different ways, and not least what it means to ourselves, if the person moves toward us or from us, and with what force and speed the person moves. Many natural phenomena consist of movement: of force, space, time, and direction/intention. Winds and waves have power and direction, as does the movement of the sun and moon across the sky. The seasons also consist of movement, a clear change over time. This also includes the dynamics of winter cold and summer heat. Vitality can be interpreted both emotionally and cognitively; Stern (2010) claims. The forms of vitality can be described as “an extension of holistic thinking (…) They unite the elements that put flesh on experience (…) because our minds tend to see dynamic events in terms of vitality forms whether they come from nature, self, or other humans. The dialogue between external and subjective reality never stops.” Theories of Gestalt were developed in the field of perception psychology (Ellis, 1938; Wertheimer, 1938) and imply that our brains strive to generate wholes, instead of just collecting simple, unrelated elements. When the perceptual system has created a Gestalt, it constitutes its own reality, regardless of the parts: “the whole is something else than the sum of its parts” (Brownell, 2016). Stern (2010) argued that movement connects phenomena to a Gestalt, which we can perceive. Movements and changes in nature can thus, together with trees, animals, formations in the landscape, etc., be perceived in the form of wholes, Gestalts, or archetypes.

By experiencing the Gestalt in its context, a person can understand its importance and be able to make the right decisions: a phenomenon that also has been further developed in therapies (Perls et al., 1951; Brownell, 2016; Rhyne, 2016). This can be compared to what Jung (1960) referred to as the transcendent function. It just appears, comes from within us, and overwhelms us with an understanding which can bridge the conscious and the subconscious, striving for wholeness and meaning (Knox, 2004). Knox (2004) argues that this wholeness is interpreted by humans as an archetype, which has the power to make us find solutions. Hence, the archetype can play a decisive role in a person’s growth, change, and development, in which mental contents emerge from the interaction of congenital functions, the brain and the external environment (Knox, 2003, 2004). We suggest that movements, changes, dynamics, and sensory impressions in natural environments create the wholes we characterize as nature archetypes. Depending on their content and information, they activate different psychological and physiological reactions. People, e.g., become worried when walking through a dark forest or through desolate landscapes, especially in storm or fog. On the other hand, experiencing soft waves rolling toward a beach, walking along winding paths or resting in a hammock, and listening to the chirping of birds soothe people. Experiences from nature-based interventions, such as in Alnarp Rehabilitation Garden, show that it is important that participants experience security and calmness (Sahlin et al., 2012; Pálsdóttir et al., 2014). The possible involvement of oxytocin in these calming effects will be discussed in section “Calm and Connection, and Its Relation to the Oxytocinergic System.”

#### Affinity and Attunement Are Needed to Start the Process of Instoration

The clusters we described above (Figure 1) consist of experiences of visual impressions, temperature, smells, and sounds that together form units. The point of departure for our argumentation about instorative effects is that people experience the world through their bodies, with all their senses and where most of their experiences occur unconsciously, subliminally (Eimera and Schlaghecken, 2003; Kiesel et al., 2007; Blakemore et al., 2017). Scents, temperature, sounds, and other sensory impressions are found to be crucial if a human being feels security and confidence in the environment, or the opposite, finds discomfort and threats (Fontaine et al., 2001; Appukuttan, 2016). Environments send different kinds of subtle signals, which might be interpreted as creating a secure atmosphere or not. Through embodied cognition, sensory experiences of impressions from the environment can present a sense that can guide further actions (Gibson, 1979; Balcetis and Cole, 2009; Grahn et al., 2010; Ottosson et al., 2015; Adevi et al., 2018). One result in this study was the key category of being able to build trust and friendship, with oneself and with nature. It is about creating a sense of affinity for a place in the natural environment. The possible role of oxytocin for the development of affinity for a natural place will be discussed in section “Calm and Connection, and Its Relation to the Oxytocinergic System.”

Instoration is hypothetically based on the precondition that the environment and the person are attuned. This, in turn, requires that the human being can feel secure or comfortable in the environment and be able to communicate intimately with nature. In that case, the conditions are met which allow human beings and nature to attune to each other. However, as can be seen in section “Experiences from Alnarp Rehabilitation Garden,” these self-regulating therapeutic activities do not occur immediately, nor do they occur in whichever setting. It is a process where the participants in nature-based therapy in Alnarp Rehabilitation Garden initially find it difficult to find a place, be able to take in and, even less, be able to communicate with nature (Sahlin et al., 2012; Pálsdóttir et al., 2014). After a few weeks in nature-based therapy, if certain conditions are met regarding security and trust in the place of therapy, things start to happen: Participants begin to be open to communication, they are attuned, and communication through emotions, intuition, and cognition can start (Ottosson, 2007; Grahn et al., 2010; Sahlin et al., 2012; Pálsdóttir et al., 2014; Spring, 2016).

Attunement is a process that has been described as being of fundamental importance by the psychotherapist and physician Poul Bjerre. A central concept in his theory was the “death and renewal rhythm”; that is, when something important in the person’s life must be left behind, in order for the new to be received and integrated, such that one should be able to move forward in life toward new goals (Bjerre, 1929, 2004). Bjerre claimed that affinity with nature can help in crisis management and healing. In order to succeed, the person must switch between efforts toward the goal and relaxation that can provide space for nature’s assimilation, attunement, and healing forces. It is about a shift between, on the one hand, logical analysis and a willingness to understand and solve problems and on the other hand sheer relaxation and contemplation, which gives scope for interpretation of symbols and Gestalts, and a medical process. In nature, Bjerre said, it is about finding places where you can attune. Through the attunement process, the healing process can be accelerated. The symbol formation and interpretation, according to Bjerre, is a productive effort – a continuous processing of the psyche of what meets the human being (Bjerre, 1929, 2004).

Section “Study Two: Facing Nature During Rehabilitation” contains quotes describing how Johan Ottosson increased his coping ability during his life crisis by encountering various natural phenomena. The ability to communicate with nature archetypes is probably important in everyday life. However, the importance could be of greatest value when facing life crises. When experiencing a life crisis, reality might be perceived to be like a quagmire – strange, weird, and sometimes even sinister and hostile (Moos, 1986). Could it be that nature archetypes can offer consistency and support for reflection, in order that one can move on: A prerequisite for building a starting point, a bridge between emotions and analytical thinking, which can facilitate intuition and promote how to work through the crisis? We envisage the archetypes communicate with us, where we can walk the path, face the storm, discover the power of fertility, or seek security in the archetype of eternity. It might perhaps help us manage with our life situation, make us comprehend it, and discover some form of meaningfulness, thus providing support to our coping strategies (Moos, 1986; Park and Ai, 2006). One hypothesis is that certain nature archetypes provide security and trust, where people can feel a belonging, and offering people consolation and attunement. Thenceforth different nature archetypes can provide clarity that can help people see the whole, discover opportunities and make intuitive decisions.

Nature archetypes can hypothetically create an intuition and thought that can support instoration and reorientation which will promote how to work through the crisis. Intuition is about being able to see the wholeness, to understand the context, and then being able to make decisions (George and Dane, 2016; Okoli and Watt, 2018; Remmers and Zander, 2018). Intuition is about considerations that lead to decisions, which are based on both emotion and cognition. Yet, the decisions are usually made relatively quickly. Sometimes, however, we need to think longer to consider decisions, but in these cases too, both emotion and analytical thinking are connected (Kahneman, 2011; Okoli and Watt, 2018). Rational decisions are usually largely based on intuition, more rarely on slower analytical thinking. However, when people are in crisis, rational decision-making is inhibited, and wrong decisions can be made, because people are not able to link emotions and cognition to a holistic consideration (George and Dane, 2016; Okoli and Watt, 2018; Remmers and Zander, 2018). In order to understand the outside world, one must be reasonably attuned. Through attunement, an affinity with the outside world, paired with logical analysis linked to emotions and intuition, people have a possibility to feel and understand a fact, and based on that can make informed decisions (Kahneman, 2011; George and Dane, 2016; Okoli and Watt, 2018; Remmers and Zander, 2018).

We choose to use the concept “scope of meaning” (Grahn, 1991; Grahn et al., 2010), which is a resource that relates to a person’s communication with the external environment. Experiences that build a person’s scope of meaning are bodily and sensory (visual, tactile, audibly, etc.) and are related to feelings and thoughts. It is an embodied communication, about relations to the surrounding social, cultural, and physical world (Allwood, 2008). Values are connected to people, single objects, the cultural context, and the physical environment. The scope of meaning may be seen as being made up of all experiences and values, and how the person communicates with the surrounding world in order to function and survive in it. It is an important part of how the person identifies him/herself and his/her functions in different contexts and determines how the person perceives the environment and his/her own scope of action (Grahn, 1991; Grahn et al., 2010). That is, a person’s “scope of meaning” forms the framework for and guides the person’s “internal working model.” Scope of meaning is linked to “theory of mind,” “theory of attachment,” and “theory of place attachment.” Scope of meaning is a framework that, for example, implies that people can have confidence in certain people and places and understand how other people think and act. Based on this framework, people form an internal working model, which works for both routine matters and in more complicated situations (Grahn, 1991; Riley, 1992; Delius et al., 2008; Grahn et al., 2010; Morgan, 2010; Lewicka, 2011; Young, 2011).

During a life crisis, communication between the surrounding world and the person becomes difficult. It may be, for example, that the person has become seriously sick, or that an important friend or family member in the person’s life has died (Machin, 2013). This means that the person’s entire scope of meaning changes or even collapses, which means that it needs to be rebuilt (Ottosson, 2007; Grahn et al., 2010; Stigsdotter et al., 2011). The person’s way of thinking about people, objects, and the whole environment will be transformed, which alters the person’s cognitive significations in relation to the surrounding world and his/her own inner working model. During the crisis, before the new scope of meaning is in place and everything has been more or less stabilized, people, objects, and environments that have previously been easy to interpret, felt familiar and pleasant, can suddenly seem strange and even unfriendly (Ottosson, 2007; Grahn et al., 2010).

We suggest that instoration is about the reconstruction of a person’s scope of meaning. If something important changes in a person’s scope of meaning, everything changes. When, in crisis, a person’s whole world is shaking and changing, it is difficult to get an overall picture and be able to understand what should be done (Moos, 1986; Machin, 2013). If a person no longer has any internal working model because it is destroyed, it might be difficult to find consolation and/or meaning in everyday life (Park and Ai, 2006; Klass, 2014). To be able to rebuild the scope of meaning, as a resource for the person’s new internal working model, it is necessary to have support. According to the supportive environment theory (Stigsdotter et al., 2011; Adevi, 2012; van den Bosch et al., 2018), it should start with simple, clear components in nature. People are hard to interpret and understand, and a person can easily feel alone or hurt in the presence of other people. Hence, relationships with other people are complicated to handle, while they are much simpler with nature, especially certain types of nature (Ottosson, 2007). In such cases, it is important to be able to find calm and connection in safe places in nature. Here, people in solitude can start to communicate with nature through nature archetypes.

### Calm and Connection, and Its Relation to the Oxytocinergic System

#### The Oxytocinergic System as an Underlying Factor

The core variable that was discovered in our study was calm and connection. We interpret this variable to be associated with all other key categories. The core variable can in turn be associated with the oxytocinergic system. In this paper, we will put forward the hypothesis that the oxytocinergic system plays an important role in mediating the positive mental and physiological effects which are induced when humans are staying in nature. Most of the knowledge on oxytocin has been obtained from experimental studies on animals and also from studies of social interaction between humans. We therefore base our hypothesis on the striking similarities between the mental and physiological effects observed in response to staying in nature and those induced during social interaction between humans or between humans and animals. In fact, the effect pattern in both these situations involve stimulation of social interaction and of calm and wellbeing, decreased levels of stress as well as stimulation of healing and growth: all cardinal effects of the oxytocinergic system.

Oxytocin is produced in the SON and PVN of the hypothalamus. As mentioned in the introduction, oxytocin is transported to the posterior pituitary to be released into the circulation and to act as a hormone. In addition, oxytocinergic nerves project to many important regulatory areas in the brain, where oxytocin influences behavioral and physiological functions. In this way, activation of the oxytocinergic system may give rise to integrated effect patterns. Oxytocin was originally known as the hormone that stimulates uterine contractions during birth and milk ejection during breastfeeding (Uvnäs-Moberg et al., 2020a). The role of oxytocin has with time become expanded to involve neurogenic effects in the brain which induce a multitude of behavioral/psychological and physiological effects. The core effects of oxytocin are stimulation of social interaction and wellbeing, reduction of fear and stress, and stimulation of healing or restorative effects (Uvnäs-Moberg, 2013). We propose that the oxytocinergic system may play an important role as a mediator of many of the beneficial effects of staying in nature and may also play a part in the positive and restorative effects that were summarized in this study.

This paper is not the place to list all the targets for the oxytocinergic projections and their effects, but it is important to mention a few of them which might be involved in the positive effects induced by nature: the amygdala where oxytocin exerts inhibitory actions on fear and stimulates social interaction, the PVN where oxytocin exerts inhibitory actions on stress corticotrophin releasing factor (controls the activity of the HPA axis), the noradrenergic neurons in the LC (controls the activity in the HPA axis and of the sympathetic nervous system), the autonomic nervous centers in the brain stem (inhibits the activity in the sympathetic nervous system and stimulates the activity in the parasympathetic nervous system), the periaqueductal gray, and the dorsal columns of the spinal cord, where oxytocin inhibits pain and inflammation and the reward centers where oxytocin together with dopamine induces wellbeing (Uvnäs-Moberg, 2013; Uvnäs-Moberg et al., 2020b).

#### The Oxytocinergic System and (Social) Interaction

Under normal circumstances, humans start communication by seeking eye contact with each other. Often other types of contact follow, such as verbal contact, touching, and hugging. These behaviors are linked to a sense of reward and relaxation and to a decrease of stress levels. Also bonding between the two individuals may be induced as a consequence of in particular repeated positive interaction (Auyeung et al., 2015; Takahashi et al., 2015; Andari et al., 2016; Shamay-Tsoory and Abu-Akel, 2016; Pawling et al., 2017; Uvnäs-Moberg et al., 2020b).

Oxytocin is released during social interaction between humans. Oxytocin release is, e.g., promoted by eye gaze, and according to the social salience hypothesis, certain aspects of the face, in particular the eyes, become more “attractive or interesting” to look at, under the influence of oxytocin and when the duration of eye gaze is prolonged (Theodoridou et al., 2009; Auyeung et al., 2015; Shamay-Tsoory and Abu-Akel, 2016; Hovey et al., 2020). Oxytocin is also released in response to verbal contact, touching, and hugging, and this second phase of oxytocin release is particularly linked to the anti-stress and healing effects of oxytocin. Sensory nerves are activated in response to physical touch, among those the CT afferents: an old type of sensory nerves, which are linked to the experience of wellbeing (Pawling et al., 2017). Activation of the CT fibers and also of other types of nerves originating in the skin as well as auditory, visual, and olfactory stimuli contributes to the release of oxytocin induced as a consequence of social interaction between humans (Takahashi et al., 2015).

The importance of oxytocin for social interaction between humans is further substantiated by experiments showing that administration of synthetic oxytocin *via* nasal spray facilitates proactive social behaviors and increases, e.g., eye gaze during social interaction. Moreover, administration of oxytocin increases an individual’s ability to interpret other individuals’ facial language and tone of voice (Hollander et al., 2003, 2007; Domes et al., 2014; Skuse et al., 2014), as well as body language (Kéri and Benedek, 2009).

Oxytocin also increases the sensitivity to touch, and infusions of oxytocin have been shown to increase touch induced oxytocin release (Velandia, 2012), during positive interaction and thereby to the oxytocin mediated effects (Takahashi et al., 2015). Recently, studies demonstrating that oxytocin administration promotes social interaction, positive emotions, and reward have been extended to include brain studies using fMRI. These studies have shown that administration of synthetic oxytocin enhances the activity in brain areas linked to wellbeing, reward, and social activity (Andari et al., 2016; Bethlehem et al., 2017).

The role of oxytocin is, however, not limited to interaction between humans. It also occurs in connection with interaction between humans and companion animals, for example, dogs. Eye gaze between humans and dogs, just as between humans, has been demonstrated to be linked to oxytocin release (Nagasawa et al., 2015). In addition, similar stress-reducing and health-promoting effects have been demonstrated to occur following human-animal interaction as following human-human interaction (e.g., Handlin et al., 2011). These data suggest that the role of oxytocin in promoting various aspects of relationships is not limited to human-human interaction.

The effects induced by oxytocin in humans, either in response to a natural release of oxytocin during interaction between humans or as a consequence of administration of synthetic oxytocin, as summarized above, display a striking resemblance with the effect pattern induced by nature on humans. As reported above, staying in, being exposed to, and/or interacting with nature have been shown to be linked to anti-stress effects, such as lowering blood pressure, heart rate, and cortisol levels; reduced inflammation; and increased skin conductivity and HRV (e.g., Bay-Richter et al., 2012; Payne and Delphinus, 2018; Twohig-Bennett and Jones, 2018; van den Bosch and Bird, 2018). In addition, staying in nature has been demonstrated to induce restorative and healing effects (Li et al., 2016; Grahn et al., 2017; Wen et al., 2019). Studies, where human subjects are exposed to visual and audible impressions from tranquil natural environments, also show that the heart rate variability increases as a sign of increased parasympathetic nervous activity (Annerstedt et al., 2013; Grahn and van den Bosch, 2014). In fact, this effect pattern, consisting of decreased activity of the HPA axis and of the sympathetic nervous system and increased activity in the parasympathetic nervous system, and in addition increased wellbeing, healing and restorative effects, is consistent with an activation of the oxytocinergic system (Uvnäs-Moberg et al., 2005, 2015).

We suggest that nature sceneries that transmit the experience of, e.g., beauty, security, and tranquility, activate the oxytocinergic system, just like human faces, in particular eye gaze, does. Some types of nature may trigger oxytocin release and thereby attract attention and create a wish to approach and to interact with nature in analogy with the salience hypothesis for human faces as suggested by Shamay-Tsoory and Abu-Akel (2016). Such triggers may convey not only an experience of beauty but also of safety and of access to resources of vital importance for survival. The presence of water may serve as a clear example of such an attractant and rewarding stimulus (White et al., 2010). These types of attraction to nature may be linked to the concept of biophilia and should have played a very important role during evolution in particular for humans living as hunters and gatherers.

Nature archetypes, perceived as friendly, lush, and safe, may attract humans (Ottosson and Grahn, 2021). Nature may influence humans through visual experiences, such as colors and shapes; olfactory stimuli, such as the scent of flowers or mushrooms; and auditory stimuli, such as birdsong or tranquil sounds from the wind in the treetops or from waves rolling toward the beach. In addition, tactile stimuli and temperature, such as walking in warm sand or feeling the warm wind or the sun against the skin, may together with proprioceptive and gravitational sensations play a part in the experience of the interaction with nature. All of these natural stimuli may trigger oxytocin release in humans, in analogy with the oxytocin release occurring in response to positive and pleasant stimuli of different types in connection with human-human or human-animal interaction. This assumption of course remains hypothetical until specific experiments have been performed. However, exposure to pheromones produced by plants may promote the release of oxytocin in humans. Recently, Ogata et al. (2020) were able to show that exposure to lavender fragrance led to an activation of oxytocin neurons in PVN. In addition, preliminary experimental data from a pilot study show that oxytocin levels rise and stress levels decrease in individuals who are exposed to green indoor plants (Mikkonen et al., unpublished).

#### The Oxytocinergic System: Stress-Reducing and Restorative Effects

One important consequence of social interaction is that anti-stress and restorative effects are induced as well as wellbeing. As a consequence of, e.g., non-noxious, pleasant stimulation of sensory nerves from the skin during social interaction, aspects of the oxytocin system will be activated and give rise to decreased activity in the HPA axis and in the sympathetic nervous system, as well as to increased activity in the parasympathetic nervous system and the dopaminergic mechanisms in the reward centers. When repeatedly activated, this effect pattern will become sustained and will be linked to health-promoting effects. Long-term positive human relationships as well as positive relationships between humans and their pets are associated with better health, in particular cardiovascular health. These effects have been attributed to oxytocin, which in addition to inducing immediate effects also induce long-term effects (Petersson et al., 1996; Beetz et al., 2012; Bernaerts et al., 2017).

Several animal studies have shown that exposure to nature can counteract stress. Experiments on rats in enriched environments show large differences between animals exposed to an environment enriched by natural elements, such as twigs, leaves, and logs, compared to if the environment is enriched with artificial materials, such as plastic toys and synthetic nesting materials. Measurements showed that the animals in the natural environment showed lower stress levels and better problem-solving ability (Bardi et al., 2016; Lambert et al., 2016). Exposure to the natural environments increased the ability to, for example, interpret hazards and make the right decisions. Lambert et al. (2016) interpreted the results as meaning that natural habitats gave the animals opportunities to maintain emotional health better and provide a buffer against stress. A later study showed that animals in environments enriched both socially and with natural materials led to higher oxytocin levels (Neal et al., 2018).

We propose that oxytocin released by nature in humans creates similar positive, health-promoting effects, characterized by reduced stress levels, by increased wellbeing (less depression) and also by stimulation of healing and growth. This effect may in fact underlie and explain the stress-reducing and health-promoting effects described by Ulrich (1984). Our theory, however, involves a nature-induced release of oxytocin and a consequent oxytocin-mediated decrease of stress levels. There are even studies using modern brain visualization techniques, such as fMRI, which demonstrate that experiencing natural environments really affects human brain function. Areas in the brain that are involved in appreciating and enjoying natural landscapes activate perceptual processes, cognitive processes, and the reward system (Zhang et al., 2019).

#### The Oxytocinergic System: Attachment and Bonding

One important function of oxytocin is to promote the establishment of bonding or attachment between individuals. The mother infant bond is one example of such an oxytocin dependent bond. Most studies regarding the mechanisms involved in formation of attachments or bonds have, however, been performed on monogamous voles. These animals normally spend their whole lives together as soon as bonding has occurred. Under natural circumstances, oxytocin released in response to sex underlies the formation of bonds. Injections of oxytocin into the brain of female and male voles can initiate the development of bonds between two individuals, and this effect involves an experience of enhanced salience of the sensory cues from “the other individual,” facilitated memory formation of “the other one,” activation of the reward center, and also decreased stress levels. In this way, the individual being close (“the other one”), to the one receiving oxytocin will appear attractive, will induce wellbeing and reduce stress levels, since oxytocin also facilitates the linkage between these different physiological effects. When a bond has been established, recognition of “the other one” together with the link to positive emotions will attract the two individuals to each other and separation may be linked to decreased wellbeing and increased levels of anxiety and stress (Petersson et al., 1996; Uvnäs-Moberg, 2013).

In humans, formation of bonding or attachment between two (or more) individuals (e.g., mother and infant, siblings, and couples) is facilitated and initiated by several types of oxytocin releasing sensory cues, such as skin to skin contact, eye contact, sound of voice, and pheromones. As interaction between individuals triggers other oxytocin-related effects, such as wellbeing and stress reduction, a bond may be developed between individuals. Such oxytocin linked bonds are of course also related to the concepts of attachment and secure base as formulated and described by Bowlby (Bowlby, 1982; Velandia, 2012; Uvnäs-Moberg, 2013).

Oxytocin release also occurs during contact with companion animals, and repetitive activation of the oxytocin system during interaction with the animals is suggested to be the one of the underlying mechanisms behind bonding to the animals (Handlin et al., 2011; Beetz et al., 2012; Julius et al., 2012; Nagasawa et al., 2015). We hypothesize that humans may also form relationships and bonds with natural areas that from a functional point of view resemble inter-human relationships or human-animal relationships. Several studies have been done on how children develop place attachment (Chawla, 1992; Korpela et al., 2002), and places that meant a lot while growing up can continue to be important during a large part of life, especially in life crises (Aronsson, 2004; Adevi and Grahn, 2011, 2012). When it comes to separation distress, Fried (1963) classic study shows how people miss and mourn lost places. This phenomenon is repeated in other studies from wars and natural disasters, such as when hurricane Katrina destroyed many places in the southern United States (Giuliani, 2003; Scannell and Gifford, 2014).

It is easy to assume that well-known places, which have been associated with loved ones and positive feelings, will develop into positive memories, which will then be linked to the calming and anti-stress effects of oxytocin. Such places will be attractive, and a kind of bond between humans and places with such positive qualities may be developed. Some people develop a long-term affection to nature. As suggested above, certain aspects of nature may attract humans and also be linked to oxytocin release. The aspects of nature that originally triggered oxytocin release will then be connected to the physiological effects of oxytocin characterized by decreased levels of fear, decreased stress levels, and increased wellbeing. In this way, an attachment or a bond between humans and nature may be formed (Adevi and Grahn, 2011, 2012). The stronger the bond, the stronger the feelings of relaxation, and wellbeing will be while staying in nature and in the long run, other positive restorative effects will also be stimulated that ultimately promote health.

#### The Oxytocinergic System: Link to Decreased Stress Levels and Thus Opportunities for Instorative Processes

We would like to propose the concepts of affinity and attunement to nature which would correspond to achieving a kind of “friendship” with a place in nature. In the presence of a “friendly environment,” e.g., a natural environment to which a person is attuned and has affinity to, oxytocin will be released. As a consequence of oxytocin release, fear and stress levels will be reduced, and under such circumstances, reorganization of functions in the brain will be facilitated (Beetz et al., 2012; Julius et al., 2012; Nagasawa et al., 2015; Richardson et al., 2016). This support can lead to fundamental psychological changes, which can lead to instorative mental processes. The experience of decreased fear and of increased trust with “Mother Nature” will allow deeper instorative, healing processes to occur, as in other therapeutic situations (Grahn et al., 2010; Sahlin et al., 2012; Pálsdóttir et al., 2014). An important consequence of oxytocin-induced inhibition of fear is the ability to reorganize experiences and memories. When fear is absent, it is possible to introduce new ways of thinking and even exchange emotional links to memories (Eckstein et al., 2015; Engert et al., 2016). This can very well occur in certain types of natural environments, if an individual feels safe and secure and has acquired affinity for the place. The memories of the traumatic experiences persist, but the emotional links are transformed from being very stressful to being calming (Ottosson, 2007; Grahn et al., 2010).

When a person feels a deep calmness and security, opportunities arise to be able to handle and understand his/her own thoughts and feelings. When this happens, opportunities may arise to be able to give up thought patterns and thus find new ways. It is therefore possible that changing of coping strategies or reorientation occurs in such environments. In this way, particular places in the nature, which are experienced as calming or familiar by an individual, may serve in analogy to social support in relationships. That is, the presence of another supportive human exerts a stress-reducing effect, which decreases fear and instead opens for reorientation and progress. For people who have experienced a lack of secure attachment and object constancy to persons, nature may be even more advantageous. In fact, individuals with insecure attachments may experience good consolation by companion animals and/or places in nature, since they do not have any memories of inconstant and insecure emotional attachment to these objects (Grahn et al., 2010; Sahlin et al., 2012; Pálsdóttir et al., 2014).

## Closing Words

In this article, we have presented a hypothesis that needs to be tested. We are aware that many studies are needed that can support different stages in the statements we have made, regarding both restorative and instorative effects. We are also aware that several other psychophysiological reactions and patterns are involved.

In our presentation, we have chosen not to discuss the Attention Restoration Theory (Kaplan, 1995, 2001), which is about how people’s ability to direct attention can be restored in certain types of natural areas. There are also other theories about how staying in gardens and natural areas can support human health and wellbeing (van den Bosch and Bird, 2018), such as inhaling phytoncides (Li, 2012) or exposure to certain types of micro-organisms that can help our immune system (Stamper et al., 2016; Robinson et al., 2018). These theories may well have an impact on human health and behavior, as well as physical activity and synthesis of vitamin D through the sun’s UVB radiation (Nilsson et al., 2019). However, we are convinced that the sensory impressions of the natural areas, its archetypal appearance, have a major impact on our behavior and wellbeing, as well as affecting people’s archaic, oxytocinergic system.

## Data Availability Statement

The original contributions presented in the study are included in the article/supplementary material, further inquiries can be directed to the corresponding author.

## Author Contributions

All authors listed have made a substantial, direct and intellectual contribution to the work, and approved it for publication.

### Conflict of Interest

The authors declare that the research was conducted in the absence of any commercial or financial relationships that could be construed as a potential conflict of interest.
